# Saliva as an alternative source of high yield canine genomic DNA for genotyping studies

**DOI:** 10.1186/1756-0500-2-219

**Published:** 2009-10-29

**Authors:** Katherine Mitsouras, Erica A Faulhaber

**Affiliations:** 1 College of Osteopathic Medicine of the Pacific, Western University of Health Sciences, Pomona, CA, USA; 2 College of Veterinary Medicine, Western University of Health Sciences, Pomona, CA, USA

## Abstract

**Background:**

The domestic dog presents an attractive model system for the study of the genetic basis of disease. The development of resources such as the canine genome sequence and SNP genotyping platforms has allowed for the implementation of canine genetic studies. Successful implementation of such studies depends not only on the quality of individual DNA samples, but also on the number of samples obtained. The latter can be maximized using a non-invasive DNA collection method that can increase study participation. We compared the DNA yield and quality obtained from blood and buccal swabs to those obtained using a non-invasive saliva collection kit (Oragene ^®^•ANIMAL kit). We also assessed the success rate of PCR amplification and genotyping accuracy of DNA isolated using these collection methods.

**Findings:**

Comparison of DNA yields from matched saliva, blood and buccal swab samples showed that yields from saliva were significantly higher than those from blood (p = 0.0198) or buccal swabs (p = 0.0008). Electrophoretic analysis revealed that blood and saliva produced higher quality DNA than buccal swabs. In addition, a 1.1-kb PCR fragment was successfully amplified using the paired DNA samples and genotyping by PCR-RFLP yielded identical results.

**Conclusion:**

We demonstrate that DNA yields from canine saliva are higher than those from blood or buccal swabs. The quality of DNA extracted from saliva is sufficient for successful amplification of a 1.1-kb fragment and for accurate SNP genotyping by PCR-RFLP. We conclude that saliva presents a non-invasive alternative source of high quantities of canine genomic DNA suitable for genotyping studies.

## Background

The domestic dog (*Canis familiaris*) has emerged as a model organism to investigate the genetic basis of both normal and pathological traits. Due to controlled breeding practices within breed clubs, modern breeds are closed gene pools, with low levels of genetic variation within each breed [1]. This is in contrast to human populations where levels of genetic variation are high, rendering the identification of disease genes a challenge [1]. The genetic structure of the dog, combined with the number of genetic disorders shared among canines and humans make the dog an ideal system to study the genetic basis of disease [2]. Furthermore, sequencing of the canine genome, completion of the canine SNP map, and the development of high-throughput canine genotyping platforms, such as the Affymetrix canine SNP array (Affymetrix, Santa Clara, CA, USA) have resulted in the creation of the same technological platforms that accelerated discovery in the human genome [2]. This, in turn, has created the need for a canine sample collection method that yields sufficient quantities of high quality genomic DNA that will perform well in downstream applications.

Currently, canine genomic DNA can be isolated from a variety of samples, including whole blood, toenail trimmings or buccal cells [3]. Whole blood is a preferred source of high quality genomic DNA and provides sufficient quantities for large-scale genotyping studies [4]. However, obtaining a blood sample requires trained personnel and the invasiveness of the procedure can deter dog owners from participating in a research study. The collection of buccal epithelial cells using swabs is a non-invasive alternative, however it presents some disadvantages. Extracted DNA can contain high fractions of bacterial DNA, which can affect the quality of large-scale genotyping studies [5]. Additionally, both the yield and quality of DNA are typically lower than those from blood samples, thereby prohibiting the successful implementation of genetic studies, particularly those involving a large number of markers [4]. Finally, DNA yields can be poor when samples are self-collected, as is the case with samples collected by dog owners themselves [6].

The availability of a commercial kit for saliva collection from human subjects (Oragene ^®^DNA kit, DNA Genotek Inc, Ontario, Canada) has allowed the use of saliva as an alternative source of genomic DNA for genetic epidemiological studies [7-9]. Previous studies have demonstrated that the quantity, quality and genotyping success rate of human genomic DNA isolated using this method is comparable to that of DNA isolated from blood [7,8]. However, the response rate for saliva samples is higher than that for blood, which suggests that saliva is a preferred alternative for DNA collection in human epidemiological studies [7,9]. We sought to compare the DNA yield, quality, PCR amplification and genotyping success of two well-established methods for sample collection from dogs (blood and buccal swabs) to that of a recently introduced, commercially available canine saliva collection kit (Oragene ^®^•ANIMAL kit, DNA Genotek Inc, Ontario, Canada).

## Methods

We obtained matched saliva, blood and buccal swab samples (dogs 1, 2 and 10) or matched saliva and buccal swab samples (dogs 3-9 and 11-15) from 15 animals. Collection protocols were approved by the Western University Institutional Animal Care and Use Committee. Blood was drawn into EDTA tubes and DNA was isolated on the same day from 0.1 mL blood using the DNEasy Blood and Tissue kit (Qiagen, Valencia, CA, USA). Two buccal swabs (Isohelix T-swabs, Cell Projects, Kent, UK) per animal were collected as directed by the manufacturer, and kept frozen until purification [10]. DNA was isolated from the pooled swabs using the QIAamp DNA Mini kit (Qiagen, Valencia, CA). Saliva was collected using Oragene ^®^•ANIMAL kits (DNA Genotek, Ontario, Canada) as directed by the manufacturer [11,12]. Saliva was collected from each animal using two saliva sponges, which were placed into a tube containing Oragene ^®^•ANIMAL solution. The Oragene ^®^•ANIMAL solution/saliva samples were mixed and stored at room temperature for an average of 2 days prior to DNA purification. DNA was purified from the entire volume of Oragene ^®^•ANIMAL/saliva sample obtained from each animal using the manufacturer's protocol [11,12]. All DNA samples were quantitated using a Nanovue spectrophotometer (GE LifeSciences, Piscataway, NJ, USA) and stored in -20°C. DNA yields obtained from matched saliva, blood and buccal swabs were analyzed by paired t-test (Table 1). In order to allow direct comparisons between the different collection methods, total DNA yields were normalized by the amount of input used for DNA purification as follows: blood samples μg DNA per 0.1 mL blood, buccal swabs μg DNA per swab and saliva samples μg DNA per 0.25 mL Oragene ^®^•ANIMAL solution/saliva as suggested by the manufacturer (Table 2) [13]. The purity of each DNA sample was assessed using the A260/A280 ratio (Table 3).

**Table 1:** Comparison of the total DNA yields by collection method

	Total DNA Yield (μg)				
	***Oragene ^®^• ANIMAL***	***Buccal Swabs***	***Whole Blood***	***Fold Difference (Oragene *^®^•** ***ANIMAL/Buccal)***	***Fold Difference (Oragene *^®^•** ***ANIMAL/Blood)***

Dog 1	27.68	1.32	3.04	20.97	9.10
Dog 2	22.28	1.26	2.18	17.68	10.22
Dog 3	19.13	1.10		17.47	
Dog 4	20.63	3.38		6.11	
Dog 5	12.30	1.37		9.01	
Dog 6	8.62	0.57		15.12	
Dog 7	67.55	0.63		107.22	
Dog 8	6.25	1.22		5.14	
Dog 9	6.49	1.13		5.74	
Dog 10	16.80	0.39	2.01	43.08	8.34
Dog 11	13.13	0.50		26.52	
Dog 12	4.74	0.39		12.15	
Dog 13	4.02	0.63		6.37	
Dog 14	34.30	0.50		69.29	
Dog 15	17.50	0.30		58.33	
***Average***				**28.01**	**9.22**
***P-value***	**0.0008 **^1^	**0.0198 **^2^			

^1 ^comparison of total DNA yields obtained from Oragene ^®^•ANIMAL kit and buccal swabs by paired t-test

^2 ^comparison of total DNA yields obtained from Oragene ^®^•ANIMAL kit and whole blood by paired t-test

**Table 2:** Comparison of the normalized DNA yields by collection method

Normalized DNA Yield (μg)			
	***Oragene*^® ^•*ANIMAL*^1^**	***Buccal Swab*^2^**	**Whole Blood^3^**

Dog 1	3.46	0.66	3.04
Dog 2	2.78	0.63	2.18
Dog 3	3.19	0.55	
Dog 4	3.44	1.69	
Dog 5	2.05	0.68	
Dog 6	1.44	0.29	
Dog 7	3.75	0.32	
Dog 8	1.56	0.61	
Dog 9	1.62	0.57	
Dog 10	2.80	0.20	2.01
Dog 11	2.19	0.25	
Dog 12	1.19	0.20	
Dog 13	1.00	0.32	
Dog 14	3.43	0.25	
Dog 15	2.92	0.15	
***Average***	**2.45**	**0.49**	**2.41**

^1 ^amount of DNA obtained per 0.25 mL Oragene ^®^•ANIMAL solution/saliva sample

^2 ^amount of DNA obtained per swab

^3 ^amount of DNA obtained per 0.1 mL whole blood

**Table 3:** Comparison of DNA purity by collection method

DNA Purity #(A260/A280)			
	***Oragene *^®^•** ***ANIMAL***	***Buccal Swab***	***Whole Blood***

Dog 1	1.70	2.15	1.16
Dog 2	1.93	1.97	1.56
Dog 3	1.55	1.91	
Dog 4	1.36	1.86	
Dog 5	1.61	1.59	
Dog 6	1.92	1.85	
Dog 7	1.66	1.29	
Dog 8	1.63	1.92	
Dog 9	1.75	1.55	
Dog 10	1.55	2.17	2.04
Dog 11	1.39	1.28	
Dog 12	1.59	1.19	
Dog 13	1.60	1.21	
Dog 14	1.75	1.05	
Dog 15	1.71	1.01	
***Average***	**1.65**	**1.60**	**1.58**

**Figure 1:**
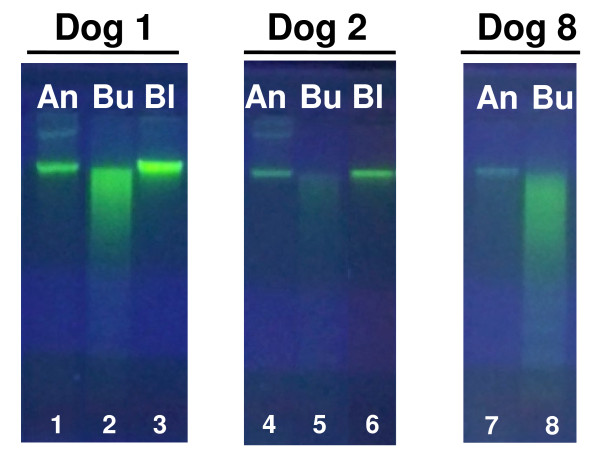
**Quality of genomic DNA extracted from paired saliva, buccal swabs and blood samples**. 250 ng of canine genomic DNA isolated using the Oragene ^®^•ANIMAL kit (An; lanes 1, 4 and 7), buccal swabs (Bu; lanes 2, 5 and 8) or blood (Bl; lanes 3 and 6) from paired samples from 3 dogs were resolved by agarose gel electrophoresis and visualized by staining with SYBR^® ^Green.

The quality of genomic DNA from a subset of the paired samples was evaluated by gel electrophoresis (Figure 1). 250 ng DNA isolated using the Oragene ^®^•ANIMAL kit, buccal swabs and blood (dogs 1-2 and 8) were resolved on a 0.8% agarose/0.5× TBE gel and stained with SYBR^® ^Green (Invitrogen, Carlsbad, CA, USA).

**Figure 2:**
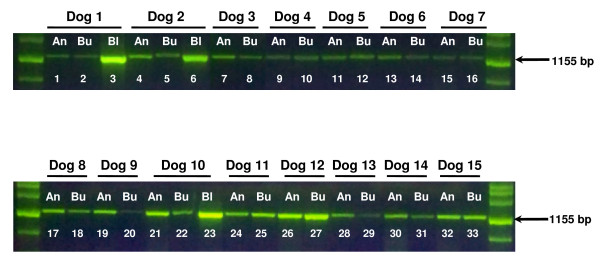
**Amplification of an 1.1-kb fragment using DNA isolated from saliva, buccal swab and blood samples**. 100 ng of canine genomic DNA isolated using the Oragene ^®^•ANIMAL kit (An; lanes 1, 4, 7, 9, 11, 13, 15, 17, 19, 21, 24, 26, 28, 30 and 32), buccal swabs (Bu; lanes 2, 5, 8, 10, 12, 14, 16, 18, 20, 22, 25, 27, 29, 31 and 33) or blood (Bl; lanes 3, 6 and 23) were used for PCR amplification of an 1155-bp fragment in the coding region of the canine transferrin receptor gene. Reaction products were resolved by agarose gel electrophoresis and visualized by staining with SYBR^® ^Green.

DNA extracted from the 15 sets of paired samples was used as a template for PCR amplification of an 1155-bp fragment in the coding region of the canine transferrin receptor gene [Genbank: 50978811] (Figure 2). 100 ng of each DNA sample were amplified using forward primer 5'-TCTCTGTGTGTGACTACCATAAATAAA-3' and reverse primer 5'-CACATAGATCTTCAAGTTCACAAA-3'(Operon, Huntsville, AL, USA). Amplification reactions were performed in a 50 microliter volume using 0.4 μM each primer, 0.4 mM dNTPs, 3.5 mM MgCl_2_, 2.5 U Taq polymerase (Qiagen, Valencia, CA, USA) in a Veriti™ 96-well thermal cycler (Applied Biosystems, Foster City, CA, USA) using the following conditions: 96°C 10 min, 30 cycles of 96°C 30 sec, 58°C 30 sec, 72°C 30 sec, followed by a final extension for 10 min at 72°C. 25 microliters of each PCR reaction were resolved on a 1.5% agarose/1× TBE gel stained with SYBR^® ^Green (Invitrogen, Carlsbad, CA, USA). A subset of the amplification products obtained from the paired samples were purified using the Qiaex II Gel Extraction kit (Qiagen, Valencia, CA, USA) and subjected to DNA sequencing at the UCLA Sequencing Core (Los Angeles, CA, USA) using primer 5'-ACTGTCCTTCTGCCTGGGAAATAGA-3' (Operon, Huntsville, AL, USA) to verify their identity.

**Figure 3:**
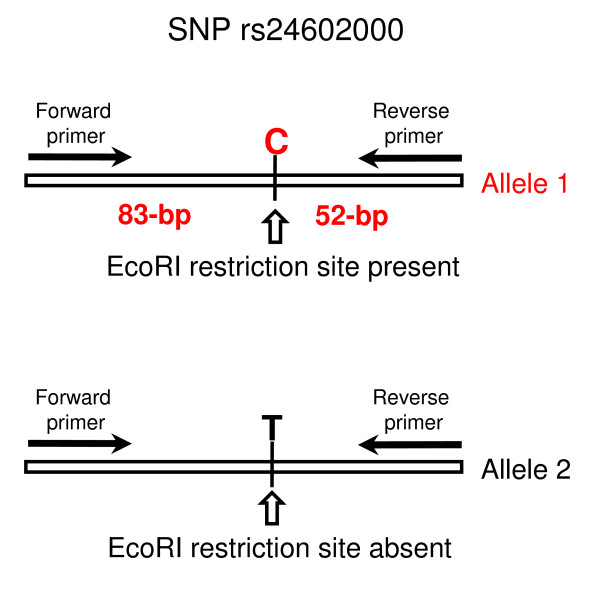
**PCR-RFLP assay for SNP genotyping**. Schematic representation of the PCR-RFLP assay used for genotyping a biallelic SNP [entrezSNP: rs24602000] in the canine serotonin transporter gene (SLC6A4). A 135-bp fragment encompassing the SNP is amplified by PCR and subsequently digested with the EcoRI restriction endonuclease. The C allele creates the EcoRI restriction site, generating two restriction fragments (83 and 52-bp). The T allele removes the restriction site and cannot be cleaved by EcoRI.

Genotyping was performed using a PCR-RFLP assay for a biallelic SNP [EntrezSNP: rs24602000] in the coding region of the canine serotonin transporter gene (SLC6A4). 100 ng of each DNA sample were amplified using forward primer 5'-CTTCCCTGAGAGTCCAGCAC-3' and reverse primer 5'-GGAGGCCCCATATTCTGAGT-3' (Operon, Huntsville, AL, USA). Amplification reactions were performed in a 50 microliter volume using 0.4 μM each primer, 0.4 mM dNTPs, 2.5 mM MgCl_2_, 2.5 U Taq polymerase (Qiagen, Valencia, CA, USA) in a Veriti™ 96-well thermal cycler (Applied Biosystems, Foster City, CA, USA) using the following conditions: 96°C 10 min, 30 cycles of 96°C 30 sec, 60°C 30 sec, 72°C 30 sec, followed by a final extension for 10 min at 72 °C. Fifteen microliters of each reaction were digested with 7.5 Units EcoRI (Promega, Madison, WI, USA) at 37°C for 2 hours. Digestion products were resolved on a 2.5% agarose/1× TBE gel and stained with SYBR^® ^Green (Invitrogen, Carlsbad, CA, USA). The C allele creates the recognition site for EcoRI, resulting in cleavage of the 135-bp PCR product into two fragments (83 and 52-bp; Figure 3).

## Results

Table 1 shows the amounts of DNA obtained using the Oragene ^®^•ANIMAL kit, buccal swabs or blood from 15 dogs. The DNA yields from saliva were significantly higher than those from buccal swabs (p = 0.0008, paired t-test) or blood (p = 0.0198, paired t-test). Saliva yielded approximately 28-fold more DNA than buccal swabs (range 5.14-107.22-fold) and 9-fold more DNA than blood (range 8.34-10.22 fold). The total yield of each method was normalized by the amount of input sample used for DNA extraction (Table 2). The average normalized DNA yields were 2.45 μg/0.25 mL for saliva (range 1.00-3.75), 0.49 μg/swab (range 0.15-1.69) for buccal samples and 2.41 μg/0.1 mL for blood (range 2.01-3.04). Both the total and normalized DNA yields we obtained using the Oragene ^®^•ANIMAL kit are consistent with those reported by the manufacturer (average yields: 18.75 μg in present study and 11.6 μg by DNA Genotek; normalized yields: 2.45 μg/0.25 mL in present study and 1.45 μg/0.25 mL by DNA Genotek) [13]. The amount of canine DNA obtained using the Oragene ^®^•ANIMAL kit is approximately 10-fold lower than that of human DNA using the Oragene ^®^•DNA kits (11.6 μg and 110 μg respectively) and is due to the fact that the two kits represent different saliva collection platforms [13,14].

The DNA purity, as assessed by the A260/A280 ratio was comparable for all three methods, and ranged from 1.36-1.93 for saliva (average 1.65), 1.01-2.17 for buccal swabs (average 1.60) and 1.16-2.04 for blood (average 1.58; Table 3).

The quality and integrity of a subset of the paired samples was evaluated by agarose gel electrophoresis (Figure 1). The genomic DNA obtained from paired blood and saliva samples (dogs 1-2) showed uniform migration as a high molecular weight band, consistent with high-quality, intact DNA. In contrast, buccal samples were either not visible (dog 2), or migrated as a smear over a broad range of lower molecular weights (dogs 1, 3), which is indicative of DNA degradation.

The performance of the extracted DNA in downstream applications was evaluated by the amplification success rates of two different PCR assays. Since DNA degradation can adversely affect PCR amplification, we tested the paired DNA samples for amplification of an intermediate length fragment, a 1.1-kb segment of the coding region of the canine transferrin receptor (Figure 2). Successful amplification was observed in all the paired samples tested, as shown by the presence of an 1155-bp band. However, the amplification efficiency varied greatly among samples, with buccal samples in some cases showing lower amplification efficiency than blood or saliva DNA from the same animal (for example, compare amount of PCR product from saliva and buccal samples for dogs 2, 3, 9 and 13). Additionally, samples purified from blood amplified more efficiently than saliva samples from the same animal (see dogs 1, 2 and 10). The identity of PCR products obtained from the paired samples of a subset of animals was verified by DNA sequencing (data not shown).

**Figure 4:**
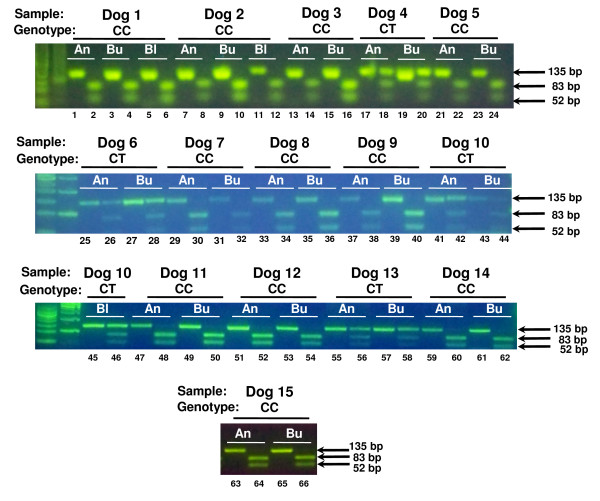
**Comparison of SNP genotyping accuracy using DNA isolated from saliva, buccal swab and blood samples**. 100 ng of canine genomic DNA isolated using the Oragene ^®^•ANIMAL kit (An), buccal swabs (Bu) or blood (Bl) were used for SNP genotyping by PCR-RFLP. A 135-bp fragment encompassing the SNP was amplified by PCR from the paired samples from 15 dogs. PCR reactions were subsequently digested with EcoRI to discriminate between the two alleles (C/T). The genotype of each animal is inferred from the pattern and size of DNA fragments obtained after digesting with EcoRI, resolving the reactions by agarose gel electrophoresis and staining with SYBR ^® ^Green. Presence of the 83-bp and 52-bp restriction fragments is consistent with the C allele, whereas presence of the 135-bp undigested fragment is consistent with the presence of the T allele. Odd-numbered lanes contain undigested PCR product, and even-numbered lanes contain PCR product digested with EcoRI. The genotype of each dog is also indicated.

As an additional, independent measure of DNA quality, we used a PCR-RFLP genotyping assay for a biallelic SNP in the coding region of the canine serotonin transporter gene (Figure 3). This assay allows for rapid SNP typing, since the presence of the C allele creates the recognition site for EcoRI, resulting in cleavage of the 135-bp PCR product in two smaller fragments (83 and 52-bp; figure 3). We subjected genomic DNA isolated from the paired samples to PCR amplification, followed by digestion with EcoRI, and resolved the undigested and digested products by agarose gel electrophoresis (Figure 4). The genotype of each animal was inferred from the pattern and size of digestion products, and genotypes obtained from each set of paired samples were tested for concordance. Although the amplification efficiency sometimes varied for paired samples (see for example the saliva and buccal samples for dogs 7 and 10), we were able to obtain readable genotypes for all sets of paired samples, and the genotypes obtained from saliva, blood and buccal swab DNA were 100% concordant for all animals tested.

## Conclusion

Our findings demonstrate that canine saliva collected using the Oragene ^®^•ANIMAL kit results in significantly higher DNA yields than those obtained from blood or buccal swabs. In addition, genomic DNA purified from saliva is of higher quality than buccal DNA, and can be used to successfully amplify PCR fragments of intermediate length and for accurate SNP genotyping by PCR-RFLP. Taken together with the non-invasiveness, ease of collection relative to blood, and low bacterial content relative to buccal swabs [13] our results suggest that saliva is an alternative and ideal source of high quality DNA for canine genotyping studies.

## List of Abbreviations

DNA: Deoxyribonucleic acid; SNP: Single nucleotide polymorphism; PCR: Polymerase chain reaction; Kb: Kilobase; RFLP: Restriction fragment length polymorphism; Bp: Basepair.

## Competing interests

The authors declare that they have no competing interests.

## Authors' contributions

KM: Study design, isolated and tested DNA from the samples, performed the tranferrin receptor PCR and SNP genotyping assay and manuscript writing. EAF: Participated in study design, identified the dogs for the study, collected saliva and buccal swab samples and manuscript writing. All authors have read and approved the final manuscript.
